# Impact of Catheter-Drawn Blood Cultures on Patient Management: A Multicenter, Retrospective Cohort Study

**DOI:** 10.1093/ofid/ofae339

**Published:** 2024-06-17

**Authors:** Rebecca Wales, Winston McCormick, Andrés Blanco-Di Matteo, José L Del Pozo, Phinnara Has, Leonard A Mermel

**Affiliations:** Warren Alpert Medical School of Brown University, Providence, Rhode Island, USA; Department of Medicine, Beth Israel Deaconess Medical Center, Boston, Massachusetts, USA; Division of Infectious Diseases, Clínica Universidad de Navarra, Pamplona, Spain; Division of Infectious Diseases, Instituto de Investigación Sanitaria de Navarra, Pamplona, Spain; Division of Infectious Diseases, Clínica Universidad de Navarra, Pamplona, Spain; Division of Infectious Diseases, Instituto de Investigación Sanitaria de Navarra, Pamplona, Spain; Lifespan Biostatistics Department, Providence, Rhode Island, USA; Division of Infectious Diseases, Department of Medicine, Warren Alpert Medical School of Brown University, Providence, Rhode Island, USA

**Keywords:** bacteremia, blood cultures, bloodstream infection, catheter infection, catheter-drawn culture

## Abstract

**Background:**

Nosocomial bloodstream infections associated with intravascular catheters pose significant financial burden, morbidity, and mortality. There is much debate about whether or not blood cultures should be drawn through central venous catheters, and while guidelines advocate for catheter-drawn cultures when catheter infection is suspected, there is variable practice in this regard.

**Methods:**

We performed a retrospective cohort study assessing episodes of positive catheter-drawn blood cultures with concomitant negative percutaneously-drawn cultures in tertiary care hospitals in the United States and Spain.

**Results:**

We identified 143 episodes in 122 patients meeting inclusion criteria. Thirty percent of such episodes revealed growth of potential pathogens such as *Staphylococcus aureus*. Overall, 21% of follow-up percutaneously-drawn blood cultures obtained within 48 hours revealed growth of the same microbe after an episode of positive catheter-drawn blood cultures with negative concomitant percutaneously-drawn cultures (33% when potential pathogens were isolated; 16% when common skin contaminants were isolated). Patients with cultures growing pathogenic organisms were more likely to receive targeted antimicrobial therapy and have their catheters removed sooner.

**Conclusions:**

Many episodes of positive catheter-drawn blood cultures with concomitant negative percutaneously-drawn cultures lead to growth from percutaneously-drawn follow-up blood cultures. Thus, such initial discordant results should not be disregarded. Our findings advocate for a nuanced approach to blood culture interpretation, emphasizing the value of catheter-drawn blood cultures in clinical decision making and management.

## Teaser

For episodes of positive catheter-drawn blood cultures growing potential pathogens with negative concomitant percutaneously-drawn cultures, 21% of follow-up percutaneously drawn blood cultures obtained within 48 hours grew the same microbe. Thus, finding positive catheter-drawn cultures should not be disregarded.

Intravascular catheters remain a major source of nosocomial bloodstream infections, leading to morbidity, mortality, and excess hospital cost [1]. Most catheter colonization occurs either extraluminally from skin flora at the insertion site, or intraluminally, through contamination of catheter hubs or connectors [1, 2]. The latter may become the predominant source of catheter-related bloodstream infection with prolonged duration of catheterization [1, 2].

Although the Infectious Diseases Society of America (IDSA) guidelines [3] recommend drawing blood cultures from the catheter and percutaneously if a catheter-related infection is suspected, many institutional protocols at United States (US) hospitals discourage or forbid catheter-drawn blood cultures in an effort to reduce the classification of contaminants as central line–associated bloodstream infections, thereby reducing risk of financial penalties from the Hospital-Associated Condition Reduction Program. Spanish guidelines [4], as well as a review by content experts [5], also recommend blood cultures drawn from the catheter and percutaneously when a catheter infection is suspected. Patients who grow pathogenic microorganisms from catheter-drawn blood cultures may benefit from interventions even if there is no growth from percutaneously-drawn cultures. This can indicate that the catheter is colonized, which may lead to true bloodstream infection, or that percutaneous blood cultures may have had a lower volume blood drawn, lower inoculum, or intermittent seeding of the blood from a colonized catheter [6]. Some patients may benefit from directed antimicrobial lock therapy, systemic therapy, both, or in some cases, catheter removal based on a positive blood culture from a central venous catheter (CVC) [7–9].

We sought to evaluate possible interventions and outcomes of patients with positive catheter-drawn blood cultures and negative percutaneously-drawn cultures at 2 academic tertiary care hospitals in 2 countries. We hypothesized that positive blood cultures drawn from CVCs with negative percutaneously-drawn cultures are clinically significant in some cases, potentially providing an opportunity to improve clinical outcomes.

## METHODS

This retrospective cohort study involved a 719-bed tertiary-care teaching hospital in the US (Rhode Island Hospital) and at a 300-bed university referral hospital in Pamplona, Spain (Clínica Universidad de Navarra). Inclusion criteria were patients ≥18 years of age hospitalized between 1 January 2010 and 5 December 2022 who had at least 1 positive CVC-drawn blood culture and negative percutaneously-drawn cultures collected within 12 hours of each other during a hospital stay. Medical records were reviewed to assess admission diagnosis, reason for blood cultures, blood culture results, interventions, and patient outcomes. For patients with multiple instances of positive CVC-drawn blood cultures and negative percutaneously-drawn cultures, each instance was documented as a separate event if blood cultures were drawn >48 hours apart and resulted in growth of different microorganisms. The study was analyzed based on individual episodes of the above-noted discordant blood culture results unless stated otherwise. A sensitivity analysis was restricted to only the first episode of discordant blood culture results. Clinical laboratories at both study sites used bioMérieux blood culture systems. Common skin contaminants were classified using the Centers for Disease Control and Prevention’s National Healthcare Safety Network common commensals list of skin contaminants (https://infectioncontrol.ucsfmedicalcenter.org/sites/g/files/tkssra4681/f/wysiwyg/Common_Commensals.pdf). Anonymous data from each site were statistically analyzed individually and then as a combined data set.

At both hospitals, the sites used for blood culture collection were left to the discretion of the treating clinicians. Most blood cultures were collected by nursing staff at both hospitals. The nurses at both hospitals are trained in proper blood culture collection on site and they are instructed to collect 10 mL of blood in each blood culture bottle. For rapid identification of microbes growing in blood cultures and susceptibilities, both of the hospital's microbiology laboratories use polymerase chain reaction for rapid detection of methicillin-resistant *Staphylococcus aureus* (MRSA) when Gram stains reveal gram-positive cocci, as well as identification and susceptibility testing of gram-negative bacilli noted on Gram stains. At both hospitals, there were no changes in protocols surrounding care of CVCs and there were no changes in management of patients infected or colonized with MRSA during the study period. MRSA-colonized or infected patients were routinely decolonized at the Spanish hospital but not at the US hospital. At the US hospital, all surgical patients requiring a skin incision received a single application of nasal decolonization preoperatively. The US hospital used chlorhexidine bathing for all inpatients; the Spanish hospital did so preoperatively.

Univariate distributions of all variables are reported to describe the study population. Categorical variables are presented as frequencies and percentages. Continuous variables are reported with means and standard deviations or medians and interquartile ranges. Bivariate comparisons between groups for categorical variables were conducted using Pearson χ^2^ or Fisher exact test where appropriate. Continuous variables were compared using Student *t* test or Wilcoxon rank-sum test if data were not normally distributed. For the multivariable approach, logistic regression models were used to estimate odds ratios (ORs) for mortality while adjusting for potential confounding variables. Time to blood culture positivity was log-transformed to approximate a normal distribution before using generalized linear regression models to adjust for potential confounders. All estimates from regression models are reported with 95% confidence intervals (CIs). Potential confounding variables included as covariates in the multivariable logistic regression models for site and timing of catheter removal included age, sex, body mass index (BMI), intensive care unit (ICU) admission, potentially pathogenic organism, and infectious diseases consultation. In the models where the primary exposure was a potentially pathogenic organism, we adjusted for age, sex, BMI, ICU admission, immunosuppression, and infectious diseases consultation. All tests were 2-sided, and *P* values <.05 were considered statistically significant. Analyses were conducted using Stata/MP 18.0 software (StataCorp, College Station, Texas).

## RESULTS

We identified 143 episodes involving a positive CVC-drawn culture and a concomitant negative percutaneously-drawn culture in 122 patients (Table 1, Supplementary Table 1). At the time of the initial blood culture collection, 65% of patients had fever and 26% met systemic inflammatory response syndrome criteria [10]. Thirty percent of these episodes with discordant blood cultures grew microbes other than common skin contaminants.

**Table 1.** Patient Variables by Study Site and Overall Cohort

Characteristic	Total Cohort (n = 143)	United States (n = 67)	Spain (n = 76)	*P* Value
Age, y				
Mean (SD)	50 (21)	55 (18)	45 (23)	.02^a^
Median (IQR)	54 (35–67)	59 (42–68)	49 (24–66)	
Min–Max	0.17–87	0.17–87	2–83	
Sex				
Female	68 (48)	30 (45)	38 (50)	.62^b^
BMI, kg/m^2^				
Mean (SD)	26 (8)	29 (9)	23 (5)	<.001^a^
Median (IQR)	25 (20–30)	28 (23–33)	23 (19–26)	
Min–Max	14–66	15–66	14–33	
Comorbidities				
Cardiovascular	44 (31)	39 (58)	5 (7)	<.001^b^
CKD	12 (8)	10 (15)	2 (3)	.01^b^
Metabolic	38 (27)	35 (52)	3 (4)	<.001^b^
Oncologic (Heme)	36 (25)	0 (…)	36 (47)	
Oncologic (Solid)	37 (26)	13 (19)	24 (32)	.13^b^
Solid organ transplant	3 (2)	1 (2)	2 (3)	1.00^b^
Other	66 (46)	56 (84)	10 (13)	<.001^b^
Total No. of comorbidities				
Mean (SD)	2.7 (2.5)	4.4 (2.7)	1.1 (0.37)	<.001^b^
Median (IQR)	1 (1–4)	4 (2–6)	1 (1–1)	
Min–Max	1–10	1–10	1–3	
Catheter type				
CVC	83 (58)	35 (52)	48 (63)	<.001^b^
PICC	24 (17)	21 (31)	3 (4)	
PA	25 (18)	0 (…)	25 (33)	
Other	11 (8)	11 (16)	0 (…)	
Catheter location	(n = 142)	(n = 66)	(n = 76)	
Internal jugular	80 (56)	31 (47)	49 (65)	<.001^b^
Subclavian	32 (23)	8 (12)	24 (32)	
Upper extremity	23 (16)	20 (30)	3 (4)	
Lower extremity	7 (5)	7 (11)	0 (…)	
Admission diagnosis				
Transplant	23 (16)	0 (…)	23 (30)	<.001^b^
Fever/sepsis	24 (17)	14 (21)	10 (13)	
Cancer	17 (12)	4 (6)	13 (17)	
Chemotherapy/immunotherapy	11 (8)	0 (–)	11 (15)	
Other medical	62 (43)	44 (66)	18 (24)	
Other	6 (4)	5 (8)	1 (1)	
Admission reason				
Medical care	115 (80)	55 (82)	60 (79)	<.001^b^
Surgical care	16 (11)	12 (18)	4 (5)	
Cancer treatment	12 (8)	0 (…)	12 (16)	
Ward/service type				
Medical	106 (74)	36 (54)	70 (92)	<.001^b^
ICU	31 (22)	29 (43)	2 (3)	
Surgical	6 (4)	2 (3)	4 (5)	
Immunosuppressed				
Yes	104 (73)	34 (51)	70 (92)	<.001^b^
Reason for blood culture				
Fever	89 (62)	32 (48)	57 (75)	<.001^b^
HDI	16 (11)	13 (19)	3 (4)	
Previous positive blood culture	3 (2)	0 (…)	3 (4)	
Other	22 (15)	13 (19)	9 (12)	
Leukocytosis	7 (5)	7 (11)	0 (…)	
Sepsis/shock	3 (2)	2 (3)	1 (1)	
Pretransplant	3 (2)	0 (…)	3 (4)	
On antibiotics at time of blood draw	142	66	76	
Yes	68 (48)	30 (46)	38 (50)	.62^b^
Death during hospitalization				
Yes	27 (19)	17 (25)	10 (13)	.09^b^
Potentially pathogenic organism grown from blood culture				
Yes	43 (30)	22 (33)	21 (28)	.58^b^

Data are presented as No. (%) unless otherwise indicated.

Abbreviations: BMI, body mass index; CKD, chronic kidney disease; CVC, central venous catheter; HDI, hemodynamic instability; ICU, intensive care unit; IQR, interquartile range; PA, pulmonary artery; PICC, peripherally inserted central catheter; SD, standard deviation.

^a^Wilcoxon rank-sum test.

^b^Fisher exact test.

The median time to blood culture positivity was less for cultures containing possibly pathogenic microorganisms compared to cultures that only grew common skin contaminants (15 vs 20 hours; *P* = .048; Table 2). However, a generalized linear model revealed a nonsignificant reduction in log-transformed time to positivity for potentially pathogenic organisms (adjusted β = −.30 [95% CI, −.64 to .04]). Twenty one percent of percutaneously-drawn blood cultures collected within 48 hours of initial positive CVC-drawn and negative percutaneously-drawn cultures revealed growth of the same microbe (33% when potential pathogens were isolated; 16% when common skin contaminants were isolated; Table 2). Of patients who had their CVC removed during the study period, 53% of episodes with discordant blood cultures resulted in CVC removal within 10 days of the positive CVC-drawn blood culture result.

**Table 2.** Outcomes Based on Blood Culture Growth of Potentially Pathogenic Microorganism or Common Skin Contaminants

Outcome	Growth of Microorganism From CVC-Drawn Blood Culture		*P* Value
	Common Skin Contaminant (n = 100)	Potentially Pathogenic Microorganism (n = 43)	
Time to positivity, h (CVC-drawn culture)			
Mean (SD)	30 (32)	27 (32)	.048^a^
Median (IQR)	20 (15–27)	15 (8–32)	
Min–Max	2–158	0.9–168	
Death			
Yes	21 (21)	6 (14)	.36^b^
Follow-up percutaneously-drawn blood culture obtained within 48 h			
Yes	93 (93)	40 (93)	.04^b^
Microorganism isolated from follow-up percutaneously-drawn cultures obtained within 48 h			
Yes	15 (16)	13 (33)	
Infection at another time with same microorganism			
Yes	17 (18)	12 (33)	.09^b^
CVC removed	71 (71)	33 (77)	
Timing of CVC removal			
≤6 d	25 (35)	21 (64)	.01^b^
≥7 d	46 (65)	12 (36)	
Received antimicrobial therapy after initial positive CVC-drawn blood culture			
Yes	47 (47)	36 (84)	<.001^b^
Antimicrobial therapy			
None	54 (54)	8 (19)	<.001^b^
IV	37 (37)	28 (65)	
ALT + IV	8 (8)	6 (14)	
ALT	1 (1)	0 (…)	
Oral	0 (…)	1 (2)	
Duration of antimicrobial therapy, d			
Mean (SD)	13 (12)	18 (12)	.02^b^
Median (IQR)	8 (6–15)	12 (8–28)	
Min–Max	1–42	2–42	
Duration of hospitalization, d			
Mean (SD)	31 (41)	27 (32)	.16^a^
Median (IQR)	22 (15–35)	18 (9–33)	
Min–Max	2–332	2–190	
ID consultation obtained during the hospitalization			
Yes	38 (38)	24 (56)	.07^b^
ID consultation obtained before or after positive CVC-drawn culture known			
Before	12 (31)	2 (8)	.06^b^
After	27 (69)	22 (92)	

Data are presented as No. (%) unless otherwise indicated.

Abbreviations: ALT, antibiotic lock therapy; CVC, central venous catheter; ID, infectious diseases; IQR, interquartile range; IV, intravenous; SD, standard deviation.

^a^Wilcoxon rank-sum test.

^b^Fisher exact test.

Patients with blood cultures growing a pathogenic organism were more likely to have their catheters removed within 6 days after the blood culture was obtained compared to those growing common skin contaminants (64% and 35%, respectively; *P* = .01). Furthermore, patients with isolation of a pathogenic organism in blood cultures were more likely to receive targeted antimicrobial therapy (ie, appropriate antibiotics based on antibiotic susceptibility testing) than those with blood cultures growing common skin contaminants (84% and 47%, respectively; *P* < .001). Patients with pathogenic organisms in blood cultures were also more likely to receive antimicrobial lock therapy with or without intravenous antimicrobial therapy (14% and 9%, respectively) and intravenous antimicrobial therapy (65% and 37%, respectively) (*P* < .001; Table 2). Treatment teams were more likely to obtain infectious diseases consultation for patients with CVC-drawn blood cultures that grew pathogenic microorganisms compared with those whose blood cultures only grew common skin contaminants (56% and 38%, respectively; *P =* .07). Multivariable logistic regression modeling revealed an increased association of in-hospital mortality in patients with catheters removed within 6 days of a positive catheter-drawn blood culture result compared to those removed at ≥7 days (OR, 3.98 [95% CI, 1.05–15.16]; Table 3). A sensitivity analysis restricted to the initial episode of discordant blood culture results among patients with multiple instances revealed nonsignificant differences in the adjusted models for mortality (adjusted OR, 1.06 [95% CI, .34–3.29]) and time to blood culture positivity (adjusted OR, −0.18 [95% CI, −.57 to .21]).

**Table 3.** Crude and Adjusted Models of In-Hospital Mortality and Time to Blood Culture Positivity, Adjusting for Potential Confounding

Mortality	OR (95% CI)	*P* Value	Adjusted OR (95% CI)	*P* Value
Site				
Rhode Island Hospital (United States)	2.24 (.95–5.32)	.07	1.36 (.39–4.78)^a^	.63
Clínica Universidad de Navarra (Spain)	Referent	Referent	Referent	Referent
Timing of catheter removal				
≤6 d	4.64 (1.53–14.09)	.007	4.00 (1.05–15.2)^a^	.04
≥7 d	Referent	Referent	Referent	Referent
Potentially pathogenic microorganism				
Yes	0.61 (.23–1.64)	.33	0.83 (.28–2.48)^b^	.74
Time to blood culture positivity^c^, h	β (95% CI)	*P* Value	Adjusted β (95% CI)	*P* Value
Potentially pathogenic microorganism				
Yes	−.30 (−.65 to .04)	.09	−.30 (−.64 to .04)^b^	.08

Abbreviations: CI, confidence interval; OR, odds ratio.

^a^Adjusted for age, sex, body mass index (BMI), intensive care unit (ICU) admission, potentially pathogenic organism, and infectious diseases (ID) consult.

^b^Adjusted for age, sex, BMI, ICU admission, immunosuppression, and ID consult.

^c^Log-transformed values.

## DISCUSSION

Clinical practice related to the use of CVCs for blood draws varies widely from hospital to hospital and country to country. Some institutions avoid the use of CVCs for blood culture collection entirely out of a fear of contamination. In this study, 33% of patients with a positive culture of a potential pathogen drawn from their CVC and initial negative percutaneously-drawn blood culture were found to have a positive percutaneously-drawn culture with the same microbe on follow-up blood draw. It is important for clinicians to realize that positive catheter-drawn blood cultures with unrevealing percutaneously-drawn cultures may reflect a lower volume of blood obtained from the percutaneously-drawn blood draw [6]. Nevertheless, our findings confirm the natural history of intravascular catheter infections whereby luminal colonization may be followed by seeding of the bloodstream, leading to positive percutaneously-drawn cultures [11]. As such, our findings suggest that positive catheter-drawn blood cultures in the absence of growth from percutaneously-drawn cultures may provide valuable information requiring a clinical intervention. This may be especially important when potential pathogens grow from the initial catheter-drawn blood cultures as occurred in 30% of such episodes (eg, growth of *S aureus*, *Pseudomonas aeruginosa*, *Enterobacter* species, *Klebsiella* species, and *Candida* species). Additionally, clinicians were more likely to administer targeted antimicrobial therapy and remove a CVC within 6 days of obtaining a positive catheter-drawn blood culture when a potential pathogen grew in cultures compared to likely skin contaminants. Last, mortality was greater in patients who had more rapid removal of CVCs after an episode involving positive catheter-drawn blood cultures. This may reflect the fact that sicker patients were more likely to have their CVC removed sooner than patient with a lesser degree of illness.

Previous studies suggest that blood culture contaminants may take longer to grow than true pathogens [12]. However, with newer technologies, this difference may not be evident [13]. Though we did not observe a significant difference in median time to positivity between possible skin contaminants and pathogenic organisms grown from catheter-drawn blood cultures, this may reflect rapid growth using more recently designed blood culture systems at both of our study hospitals. Additionally, our study may have been underpowered to show a difference in time to culture positivity.

This study has potential limitations. We observed patients at 2 clinical centers in 2 countries, but our findings may not reflect blood culture practices elsewhere. We did not perform a power analysis and our study may be underpowered to assess some of the outcomes we measured. However, to the best of our knowledge, our findings may be the first to assess how clinicians respond to positive catheter-drawn blood cultures with negative concomitant percutaneously-drawn cultures under nonstudy conditions. The time to blood culture positivity was the time the blood culture was placed into the automated blood culture machine until the bottle with growth was removed from the automated machine and Gram stain was completed; however, the accuracy of these data was not otherwise verified and blood culture volume per bottle was not recorded. We did not assess data regarding repeat blood cultures beyond 48 hours, so we are unsure if initial catheter-drawn cultures led to growth of the same microbes from percutaneously-drawn culture positivity at more prolonged time intervals. We did not find a difference in mortality based on finding blood culture growth of more pathogenic organisms, but our study may have been underpowered for this outcome measure.

Our findings support IDSA and Spanish guidelines recommending catheter-drawn blood cultures when catheter infection is suspected [3–5]. Infectious diseases consultation can assist clinical teams in interpretation of positive blood cultures and help determine if therapeutic interventions should be initiated [14]. Our findings suggest that positive catheter-drawn blood cultures with negative concomitant percutaneously-drawn cultures should not be disregarded since our findings revealed that 33% of follow-up percutaneously-drawn blood cultures with potential pathogens revealed growth of the same microbe. Based on our findings, we hope that this study encourages clinicians to obtain both catheter-drawn and percutaneously-drawn blood cultures when a catheter infection is in their differential diagnosis. The optimal management for patients with positive catheter-drawn cultures and negative percutaneously-drawn cultures awaits the findings of future studies that randomize patients to different management strategies to assess outcomes. Follow-up blood cultures are warranted for those patients whose clinical status has not improved.

## Supplementary Data

Supplementary materials are available at *Open Forum Infectious Diseases* online. Consisting of data provided by the authors to benefit the reader, the posted materials are not copyedited and are the sole responsibility of the authors, so questions or comments should be addressed to the corresponding author.

## Supplementary Material

ofae339_Supplementary_Data
