# Supplementary material for: Impact of Catheter-Drawn Blood Cultures on Patient Management: A Multicenter, Retrospective Cohort Study
Source: Open Forum Infect Dis. 2024 Jun 17;11(7):ofae339. doi: 10.1093/ofid/ofae339 (PMC11221777; doi:10.1093/ofid/ofae339)
Supplement: ofae339_Supplementary_Data [file ofae339_supplementary_data.docx]

**Table S1. Line list of microorganisms isolated**

| **Line organism** | **Frequency** | **Percent** |
| --- | --- | --- |
| Coagulase-negative staphylococci | 90 | 56.96 |
| *Escherichia coli* | 11 | 6.96 |
| *Klebsiella pneumoniae* | 6 | 3.80 |
| *Micrococcus* species | 6 | 3.80 |
| *Cutibacterium acnes* | 5 | 3.16 |
| *Enterococcus faecalis* | 3 | 1.90 |
| *Enterobacter cloacae* | 3 | 1.90 |
| *Hafnia alvei* | 1 | 0.63 |
| *Actinomyces odontolyticus* | 2 | 1.27 |
| *Bacillus cereus* | 2 | 1.27 |
| *Becteroides fragilis* | 1 | 0.63 |
| *Candida parapsilosis* | 2 | 1.27 |
| *Enterococcus faecium* | 2 | 1.27 |
| *Klebsiella oxytoca* | 2 | 1.27 |
| Methicillin-Resistant *Staphylococcus aureus* | 2 | 1.27 |
| *Pseudomonas aeruginosa* | 2 | 1.27 |
| *Pseudomonas stutzeri* | 2 | 1.27 |
| *Stenotrophomonas maltophila* | 1 | 0.63 |
| *Streptococcus salivarius* | 2 | 1.27 |
| *Acinetobacter junii* | 1 | 0.63 |
| *Aerococcus viridans* | 1 | 0.63 |
| *Candida famata* | 1 | 0.63 |
| *Candida krusei* | 1 | 0.63 |
| *Corynebacterium afermentans* | 1 | 0.63 |
| *Corynebacterium* species | 1 | 0.63 |
| *Granulicatella adiacens* | 1 | 0.63 |
| Moraxella species | 1 | 0.63 |
| *Parvimonas micra* | 1 | 0.63 |
| *Raoultolla planticola* | 1 | 0.63 |
| *Steptococcus intermedius* | 1 | 0.63 |
| *Serratia marcescens* | 1 | 0.63 |
| *Streptococcus anginosus* | 1 | 0.63 |
| Total* | 158 | 100.0 |

*Some patients had multiple microorganisms isolated from a single blood culture
